# Investigating the Phytochemical Constituents, Anti‐Inflammatory, and Neuropharmacological Activities of *Podocarpus neriifolius* Leaves Through FT‐IR, GC–MS, Experimental Studies, Molecular Docking, and Molecular Dynamics Simulations

**DOI:** 10.1002/prp2.70272

**Published:** 2026-06-02

**Authors:** Neamul Hoque, Md. Tashrif Rahman Tipu, Md Abul Bashar, Raju Dash, Kutub Uddin Ahamed, Md. Liakot Ali, Md. Jahirul Islam Mamun, Bakul Akter, Mohammed Kamrul Hossain, Nawreen Monir Proma

**Affiliations:** ^1^ Department of Pharmacy, Faculty of Biological Sciences University of Chittagong Chittagong Bangladesh; ^2^ Department of Pharmacy, Faculty of Biological Sciences Islamic University Kushtia Bangladesh; ^3^ Department of New Biology Daegu Gyeongbuk Institute of Science and Technology (DGIST) Daegu Republic of Korea; ^4^ InnoCORE AI‐CRED Institute Korea Advanced Institute of Science and Technology (KAIST) Daejeon Republic of Korea; ^5^ Pharmaceutical Sciences Research Division, BCSIR Dhaka Laboratories Bangladesh Council of Scientific and Industrial Research (BCSIR) Dhaka Bangladesh; ^6^ School of Pharmacy BRAC University Dhaka Bangladesh

**Keywords:** anti‐inflammatory agents, *in silico* modeling, medicinal plant, neuropharmacology, phytochemicals, *Podocarpus neriifolius*

## Abstract

*Podocarpus neriifolius* , a widely distributed medicinal plant in South and Southeast Asia, has traditionally been used to treat rheumatism and joint pain. This study aimed to investigate the phytochemical profile and therapeutic potential of the ethanolic leaf extract of *P. neriifolius* (EEPN) using integrated in vitro, in vivo, and *in silico* approaches. Phytochemical analysis via FT‐IR and GC–MS revealed the presence of steroidal glycosides, flavonoids, alkenes, aliphatic compounds, steroids, saponins, and other glycosides. In a COX inhibition assay, EEPN demonstrated notable in vitro anti‐inflammatory effects, further validated in a mouse paw edema test. In Swiss albino mice, EEPN (200 and 400 mg/kg) showed increased head dipping in HBT (43.4 ± 1.14 and 69.2 ± 1.22), where the standard showed (57.5 ± 1.01), and improved open‐arm entries and time in EPM (260.3 and 280.6 s) compared to the standard (245.2 s). EEPN produced a dose‐dependent sedative effect both in HCT and OFT. Furthermore, EEPN significantly lowered immobility time in the tail suspension and forced swimming tests, demonstrating its antidepressant effects. According to molecular docking studies, the phytochemicals in EEPN showed a strong affinity for binding to important drug target proteins linked to inflammation, anxiety, and depression. The EEPN had binding affinities of −6.6, −5.4, and −10 kcal/mol with COX2, GABA_A_, and the serotonin transporter, respectively. Protein‐ligand complex stability was validated through molecular dynamics simulations analysis. The pharmacokinetic and safety profile of the compounds in ADMET analysis showed druggable qualities within acceptable ranges, implying the possibility as potential medications in the future. However, further research is necessary to ascertain whether *P. neriifolius* leaves can be utilized as a therapeutic agent.

## 1 Introduction

Inflammation is the term used to describe the reaction of vascular tissue brought on by infections, irritants, and damaged cells [1]. Oxidation is intimately associated with inflammation: When free radicals damage cells, inflammation results [2]. In addition to initiating a signal for the healing process, inflammation is an organism's defense mechanism for eliminating harmful stimuli. The characteristic of a chemical compound combined with a therapy that can lessen inflammation is referred to as anti‐inflammatory [3]. Numerous studies now demonstrate that depression is linked to the compensatory anti‐inflammatory reflex system, cell‐mediated immunity, and a persistent mild inflammatory process, which is typified through immunoregulatory mechanisms that are negative [4, 5]. Globally, the most prevalent causes of diverse psychiatric disorders are anxiety and depression [6]. The number of people with mental illnesses has been rising daily, especially in developing countries [7]. Nearly 322 million people suffer from sadness, according to the WHO report. According to this report, anxiety disorders impact over 260 million people worldwide, or approximately 3.6% of the population [8]. Depression is a unilateral disease that affects a person's thoughts, behavior, feelings, and perception of well‐being. It is characterized by a depressed mood and reduced action resulting from minimal monoamine levels in the brain [9]. Although anxiety is a natural emotional state, if it occurs frequently, it may be regarded as a psychological disorder. Signs of anxiety linked to depression can lead to a variety of indications, such as a decline in the actual prognosis, a decrease in responsiveness to treatment or medication, and an elevated risk of suicide [10]. Recent studies have focused on natural phytomedicines in an effort to discover new herbal anti‐inflammatory compounds with no adverse side effects, addressing the health issues that traditional drugs have demonstrated [11]. Depression is commonly treated with medications like tricyclic antidepressants (TCAs), monoamine oxidase inhibitors (MAOIs), dopamine reuptake blockers, and selective serotonin reuptake inhibitors (SSRIs) [12]. There are currently several anxiolytic and antidepressant drugs available on the market. Numerous adverse effects, such as issues with sexuality, hypotension, weight gain, sleeping disturbance, physical reliance, tolerance, and cardiotoxicity, are still linked to them [13]. Several plant‐derived products have also been evaluated for their antidepressant properties, in addition to these medications [14].

Natural biomolecules are just as effective at curing illnesses as their synthetic counterparts, despite having fewer adverse effects. Recently, there has been a significant improvement in the search for substitute medication treatments for mental disorders derived from plant extracts [15, 16]. Due to their numerous pharmacological uses, one potential way to acquire therapeutics is medicinal plants that can save lives worldwide [17]. The tree *Podocarpus neriifolius* is a species of conifer in the family Podocarpaceae, a moderate‐sized tree that can grow to a height of 35–45 m. It is rarely buttressed or spurred, and the bark's color is brownish‐gray. It is by far a highly prevalent species in the genus and is grown in gardens as well as across Malesia, from Indo‐China, Thailand, Nepal, India, and to the Fiji and Solomon Islands [18]. The tree is essential for its use in regional resources and ethno‐medical uses [19]. Although the wood from this plant is used to make paper and furniture, its consumable fruits are eaten either uncooked or cooked, and its leaf decoctions are utilized in traditional medicine to treat painful joints and rheumatism [20]. Mohamed et al. demonstrated that *P. neriifolius* had the maximum concentrations of terpenoids, phytosterols, flavonoids, saponins, tannins, phlobatannins, phenolic compounds, carbohydrates, and fats according to the qualitative assessment of bioactive metabolites [21]. Its leaves and bark are also utilized for treating rheumatism, arthritis, diarrhea, dysentery, and herpes. Boiling the leaves produces leaf water for bathing [22, 23, 24]. Previous research suggests that leaves of this plant have antibacterial and anti‐proliferative qualities [21]. Despite the long‐standing ethnomedicinal use of *P. neriifolius* leaves, there is a critical lack of systematic scientific literature regarding its phytochemical characterization and pharmacological profile. This study aims to bridge this gap by providing a scientific basis for its traditional applications. Furthermore, our experimental findings are validated through *in silico* receptor‐ligand studies, including molecular docking and molecular dynamics simulations, offering a detailed mechanistic perspective on the plant's bioactivity.

To address this scientific gap, the present study utilizes FT‐IR and GC–MS analysis to characterize the phytochemical constituents of the ethanolic extract of *P. neriifolius* leaves (EEPN). The pharmacological potential of EEPN was evaluated through a wide range of in vitro and in vivo assays, specifically targeting anti‐inflammatory, anxiolytic, sedative, and antidepressant activities. Furthermore, integrated *in silico* molecular docking, molecular dynamics (MD) simulations, and ADMET analyses were conducted to elucidate the underlying mechanisms of action and establish the pharmacokinetic profile of the identified compounds.

## 2 Methods and Materials

### 2.1 Drugs and Chemicals

Carrageenan, COX (human ovine) inhibitor assay kit, acetate buffer, ethanol, and Tween‐80 were obtained from Nurjahan Scientific Limited, Chittagong, Bangladesh. The standard drugs, including aspirin, diazepam, and fluoxetine, were supplied by Square Pharmaceuticals, located in Dhaka, Bangladesh. Other reagents were provided by the Department of Pharmacy, Faculty of Biological Sciences, at the University of Chittagong.

### 2.2 Collection of the Plant and Crude Extract Preparation

Fresh *P. neriifolius* leaves were collected for this investigation from the Hill of the University of Chittagong, Bangladesh, and identified by Dr. Sheikh Bokhtear Uddin, Professor of Botany at the University of Chittagong, Bangladesh. The voucher specimen number for this plant is CU/Pharm 1027. After being cleaned with water, the collected plant samples were left to dry for 14 days in a room with sufficient ventilation but without direct sunlight. A blender was used to powder the dried samples. For 2 weeks, 550 g of the powdered leaves were macerated in 4.5 L of ethanol and kept in amber‐colored glass containers. To ensure adequate extraction, the containers were occasionally shaken. The mixture was filtered after 2 weeks. A rotary evaporator was used to evaporate ethanol to produce a concentrated extract. The resultant 40 g crude extract (EEPN) was kept for later analysis at 4°C. Ethanol was chosen as the solvent in this study due to its widespread use in extraction processes and its demonstrated capacity to effectively extract bioactive components, particularly phenols and flavonoids with significant pharmacological significance. It can dissolve and recover both polar and nonpolar compounds due to its polarity and adaptable solubility [25].

### 2.3 Fourier Transform‐ Infrared (FT‐IR) Spectroscopy and Gas Chromatography–Mass Spectrometry (GC–MS) Analysis

FT‐IR spectroscopy and GC–MS were used to analyze EEPN in accordance with standard operating procedures. The specific experimental procedures are contained in the Supporting Information.

### 2.4 In Vitro COX Inhibition Assay

The Colorimetric COX (human ovine) inhibitor Screening assay kit was used to conduct the test [26]. In summary, 150 mL of assay buffer, 10 mL of heme, 10 mL of an enzyme (COX‐1 or COX‐2), and 10 mL of plant material (1 mg/mL) make up the reaction mixture. The peroxidase element of the COX catalytic domain is used in the assay. By tracking the appearance of oxidized N, N, N, N′‐tetramethyl‐p phenylenediamine (TMPD) at 590 nm, the peroxidase activity was measured colorimetrically. As a standard medication, aspirin (acetylsalicylic acid, 1 mM) was used. The following formula was used to determine the percentage of COX inhibition:

$$\mathrm{COX}\;\text{inhibition activity}=1-\frac{T}{C}\times 100$$

where *T* = Absorbance of the inhibitor well at 590 nm and *C* = Absorbance of the 100% initial activity without inhibitor well at 590 nm.

### 2.5 Experimental Animals and Ethical Approval

The Swiss albino mice (28–35 g, 4–5 weeks, both sexes) were procured from the Animal House at the University of Chittagong. They were kept in polypropylene cages with free access to food and water at 25°C ± 2°C, 45%–55% relative humidity, and a 12‐h light–dark cycle. They underwent 1 week of acclimatization, with food withdrawn for 12 h before and during the experiments. The Animal Ethics Review Board (AERB) of the Faculty of Biological Sciences, University of Chittagong approved the protocol using approval form number AERB‐FBSCU‐20250622‐(1).

### 2.6 Experimental Design

The animals were randomly divided into four groups, each consisting of five mice (*n* = 5 per group). The negative control group was treated with only vehicle (1% tween‐80, 10 mL/kg), which was utilized to dilute the plant extract and prepare the doses. Group I (control, 1% tween‐80, 10 mL/kg), Group II (standard, 200 mg/kg aspirin), and Groups III–IV (extract 200 and 400 mg/kg) were evaluated for anti‐inflammatory activity. Groups I (control, 1% Tween‐80), Group II (standard, diazepam 1 mg/kg), and Groups III–IV (extracts at 200 and 400 mg/kg) were tested for anxiolytic and sedative activity, where Group II (standard, fluoxetine 1 mg/kg) was employed for assessing antidepressant effect. Every medication was taken orally.

### 2.7 Acute Oral Toxicity Test

In accordance with OECD guidelines, the acute oral toxicity of EEPN was assessed [27]. For this study, six different EEPN dosages—100, 250, 500, 750, 1000, and 2000 mg/kg—were employed. For 14 days, mice (*n* = 5; gender: male) were given the extract at the previously mentioned dosages, and any toxicity was noted. For the first hour, then every 4 h, and then every 24 h for the next 14 days, observations were made. A dose of 10% of the LD_50_ was considered a safe dose.

### 2.8 In Vivo Anti‐Inflammatory Activity

#### 2.8.1 Carrageenan‐Induced Mouse Paw Edema Method

Carrageenan‐induced mouse paw edema was used to measure the anti‐inflammatory activity [28]. The protocol described in Section 2.6 was followed when grouping and treating the mice. A Plethysmometer was used to measure the amount of edema at 0–5 h after carrageenan was injected into the left hind paw. The percentage inhibition of anti‐inflammatory activity was calculated using the following formula:

$$\begin{array}{c} \%\text{Inhibition of edema}\\ \;=\frac{\left(V_{t}-V_{0}\right)\;\text{control mice}-\left(V_{t}-V_{0}\right)\;\text{treated mice}}{\left(V_{t}-V_{0}\right)\;\text{control mice}}\times 100 \end{array}$$

where *V*_t_ = Volume of mouse paw edema at time *t*, and *V*_0_ = Volume of mouse paw edema at time 0.

### 2.9 Evaluation of Anxiolytic Test

#### 2.9.1 Elevated Plus Maze Method

The elevated plus maze evaluates the anxiolytic and neuroprotective effects [29]. Located 40 cm above the surface, the elevated plus maze is a plus‐shaped apparatus with two open arms measuring 25 × 5 cm and two closed arms measuring 25 × 5 × 16 cm arranged perpendicularly. The instructions in Section 2.6 were followed when grouping and treating the mice. Every mouse was positioned facing an open arm in the middle of the maze, and over the course of 5 min, the number of entries (all four paws) and the amount of time spent in open and closed arms were noted [30].

#### 2.9.2 Hole‐Board Test Method

An installation known as “hole‐board equipment” features holes in the surface that allow an animal to insert its head. It's called “head‐dipping [31]^”^. The test comprises a 40 × 40 × 25 cm^3^ wooden chamber with 16 uniformly spaced 3 cm holes in the floor that are raised 25 cm above the ground so that the mice may insert their heads through them. The protocol in the Section 2.6 was followed when grouping and treating the mice. While an increase in head dipping indicates curiosity and exploratory behavior, animals in an anxious state typically avoid exploring new areas, which results in fewer head dips. After 60 min of treatment, the mice were put on the board and given 5 min to examine; during this time, the number of head dips was noted [32].

### 2.10 Evaluation of Sedative Activity

#### 2.10.1 Hole Cross Method

This activity was carried out in a chamber with wooden walls (30 cm × 20 cm × 14 cm) and no ceiling. The room is divided into two sections by a fixed wooden frame in the center. A circular hole measuring 7.5 cm in height and 3.5 cm in width was present in the wooden barrier. Mice were divided into groups and given the treatment outlined in the Section 2.6 for this test. A tally counter was used to record the number of times the mice entered the gap between the two rooms at 0, 30, 90, and 120 min over 3 min [33].

#### 2.10.2 Open‐Field Method

The test apparatus occupied approximately 0.5 m^2^ of floor space and was surrounded by a 50 cm tall wall [34]. A grid of small squares, which take turns being black and white, covers the ground. The guidelines in the Section 2.6 were followed when grouping and treating the mice. The number of squares the mice explored was recorded at 0, 30, 60, 90, and 120 min following oral therapy. Over the course of 5 min, the mice's movements were monitored using a tally counter.

### 2.11 Evaluation of Antidepressant Activity

#### 2.11.1 Forced Swimming Test (FST)

The forced swimming test was utilized in conjunction with the previously mentioned technique to evaluate the antidepressant effect of EEPN in mice [35]. The guidelines in the Section 2.6 were followed when grouping and treating the mice. Each mouse was put in a plastic container (25 × 15 × 25 cm^3^, 15 cm deep, with water kept at 25°C ± 2°C) 60 min after the treatment. Mice were prevented from touching the bottom of the cylinder or leaping out during the experiment. Along with active swimming behavior, the amount of time spent immobile—defined as floating with little movement and keeping the head above the water—was recorded. The first 2 min of the 6‐min test were used for acclimatization, and the next 4 min were utilized to measure how much time the mice spent immobile [32].

#### 2.11.2 Tail Suspension Test (TST)

Mice were grouped and treated according to the protocol described in Section 2.6 (Section 2.6). Sixty minutes after treatment, each mouse was suspended by the tail approximately 50 cm above the floor using adhesive tape placed about 1 cm from the tail tip. Antidepressant activity was assessed by measuring immobility duration, as effective agents reduce immobility time. Animals were suspended for a total of 6 min, with the first minute allowed for acclimatization and the remaining 5 min used to record immobility time [36].

### 2.12 *In Silico* Study

#### 2.12.1 Molecular Docking

For additional analysis, the chosen compounds were obtained in 3D SDF format from the PubChem database. In the PubChem database, compounds without 3D conformers were downloaded in 2D SDF format. Open Babel software [37] was then used to convert them to 3D SDF format. Before molecular docking, the mmff94 force field in PyRx software [38] was used to minimize ligand energy. The Protein Data Bank was utilized to acquire the 3D crystal structure of all the proteins in .pdb format [39]. The selected proteins are given in Table 1. Using the Discovery Studio 2021 client software, protein structure is prepared by removing all heteroatoms and water molecules. To create a neat protein structure, essential components like side‐chain geometry, deficient hydrogen bonds, and improper bond arrangement have been refined using the Swiss‐PDB Viewer application [40]. The GROMOS96 43B1 force field was implemented in this process. Molecular docking was done using PyRx software [38] with a built‐in docking tool named AutodockVina [41]. The 3D structures of proteins and all ligands were imported into PyRx software and converted to pdbqt format. Co‐crystalized ligands, which are known inhibitors, were also added to the compound library as a reference molecule. The dimensions of the grid box were (25 × 25 × 25) Å centered on the active site of each protein. The Discovery Studio 2021 software was used to visualize and interpret the molecular properties of the ligands with the lowest binding energy.

**TABLE 1:** Chosen drug‐target proteins to examine the phytochemicals from *Podocarpus neriifolius* pharmacological potential.

Pharmacological activity	Protein name	PDB ID
Anti‐inflammatory activity	Human cyclooxygenase 2 (COX‐2)	5IKR
Anxiolytic activity	Human GABA_A_	6X3T
Antidepressant activity	Human serotonin transporter	5I6X

#### 2.12.2 Molecular Dynamics Simulations

Each system was designed utilizing the System Builder program. Ligand‐receptor complexes were optimized, minimized, and any missing amino acid residues were filled in where needed using the Protein Preparation Wizard of the Schrödinger Suite 2023‐2 program [42]. We used the Desmond module of Schrödinger Suite 2023‐2 (LLC, New York, NY, USA) to conduct molecular dynamics simulations for a few chosen hits while applying the OPLS4 force field to obtain a better comprehension of the behavior of protein–ligand complexes in aqueous systems. Each complex was surrounded by a cubic periodic boundary box containing a specific solvent. Protein–ligand binding is influenced and facilitated by intermolecular interactions, and the whole system was configured to equilibrate with the solvated transferable intermolecular potential 3P (TIP3P) water model. Ten degrees in each direction was the maximum for the boundary conditions. More counter ions of Na^+^ and Cl^−^ (0.15 M) were introduced to the system to neutralize the solvated system as a whole. After that, an energy‐minimization procedure was applied to the entire system. The whole system was exposed to an NPT ensemble [43], that retained a temperature of 300 K and a pressure of 1.01325 bar before the simulation [44, 45]. Brownian dynamics were performed using the NVT ensemble at 10 k for 12 ps in stage two for the solute's heavy atoms. The third stage involved applying the NVT ensemble to the large atoms of the solute for 12 ps, while maintaining a temperature of 10 K. The fourth stage then utilized NPT, except NVT, where 12 ps simulations were carried out at 1 bar of pressure. In stage five, a brief simulation lasting 12 ps was performed to solvate the protein cavity. Stages six and seven were used for a 12‐ps simulation under the NPT ensemble before the final simulation, with stage seven being carried out without any restrictions on the heavy atoms of the solute. With a total of 2000 frames and a 100 ps gap between each frame, the final simulation ran for 200 ns. Following the simulation, trajectories were moved to Schrödinger2023‐2 for analysis of RMSD, RMSF, and intramolecular interactions for every complex.

#### 2.12.3 In‐Silico ADME/T Prediction

A drug candidate's safety and effectiveness primarily rely on the absorption, distribution, metabolism, and excretion (ADME) specifications, as well as distinct toxicities (T). Hence, the assessment of ADME/T properties was performed using Lipinski's rule of five [46]. To evaluate the chemical compounds' druggability, several accepted procedures were used [47]. SwissADME [48] a widely used computational server, was used to evaluate the chosen active compounds, and pkCSM [49] to identify their possible characteristics.

### 2.13 Statistical Analysis

A mean ± standard error of the mean (SEM) was used to display the data. A one‐way ANOVA and a post hoc Dunnett's test for parallels were conducted using the “Statistical Package for the Social Sciences” (SPSS, Version 16.0, IBM Corporation, New York) for statistical inspection. The following significance levels were established when comparing the results to the control group: *p < 0.05, **p < 0.01, and ***p < 0.001, indicating statistical significance.

## 3 Results

### 3.1 GC–MS Findings

**FIGURE 1:**
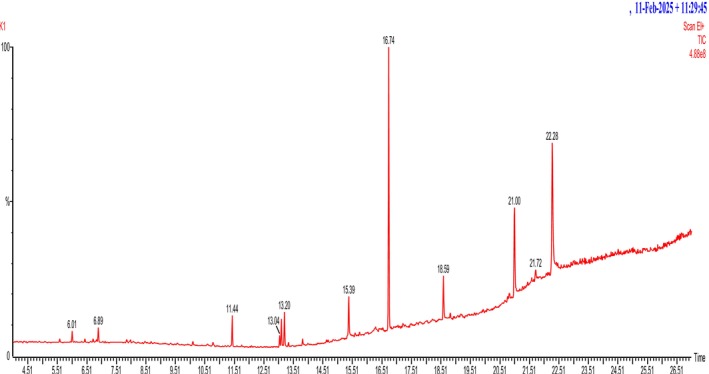
The GC–MS chromatogram of the ethanolic extract from the leaf of *P. neriifolius* .

**FIGURE 2:**
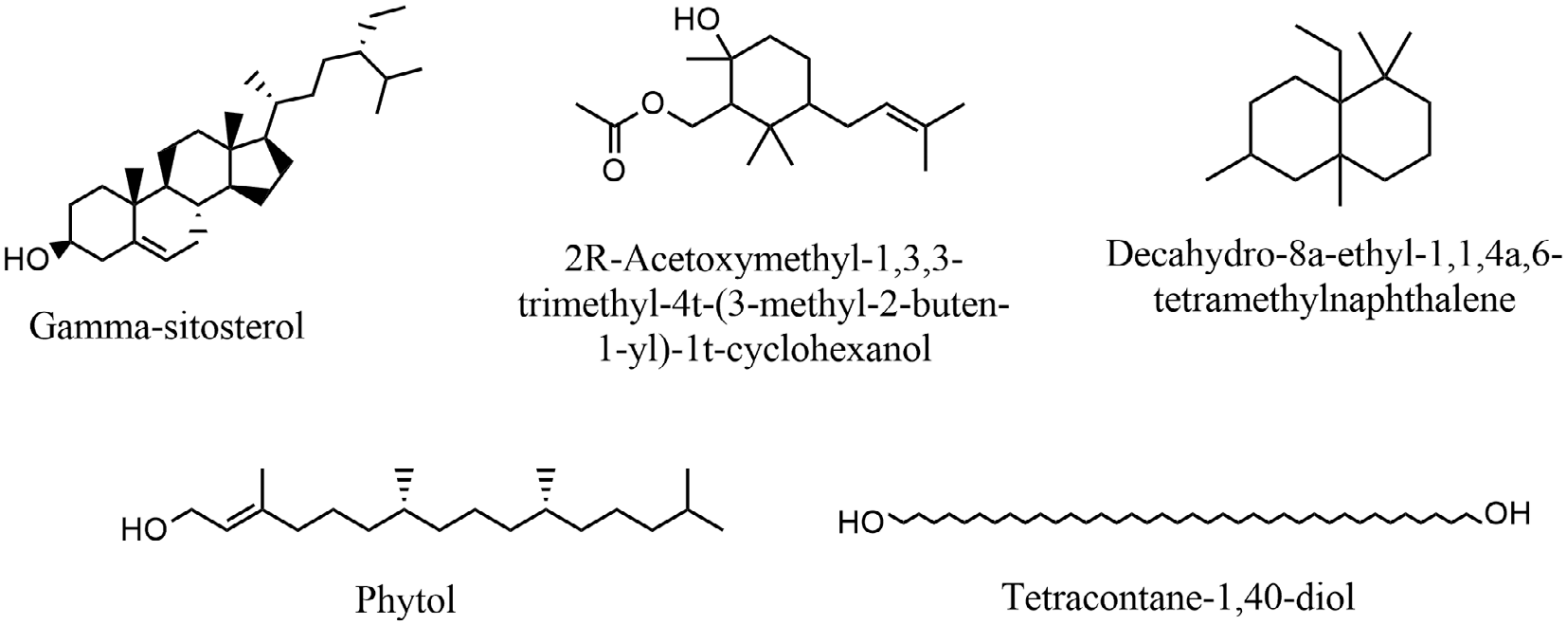
2D illustrations of the compounds detected in the ethanolic extract of *Podocarpus neriifolius* leaves using GC–MS analysis.

The ethanolic extract of *P. neriifolius* leaves (EEPN) contained seven different phytocompounds, as determined by GC–MS analysis. In Figure 1, the chromatogram is displayed. The identified compounds are listed in Table 2, together with chromatographic information and molecular details. Structures of the identified compounds are illustrated in Figure 2.

**TABLE 2:** Phytocompounds detected by GC–MS screening in the ethanolic extract of the leaf from *Podocarpus neriifolius* .

Name	M.W.	Formula	RT	Similarity
2R‐acetoxymethyl‐1,3,3‐trimethyl‐4t‐(3‐methyl‐2‐buten‐1‐yl)‐1 t‐cyclohexanol	282	C_17_H_30_O_3_	6.89	86.8
Tetracontane‐1,40‐diol	594	C_40_H_82_O_2_	10.10	74.0
Phytol	296	C_20_H_40_O	13.20	86.0
Decahydro‐8a‐ethyl‐1,1,4a,6‐tetramethyln	222	C_16_H_30_	18.56	74.3
Gamma‐sitosterol	414	C_29_H_50_O	22.28	78.7

### 3.2 FT‐IR Analysis

**FIGURE 3:**
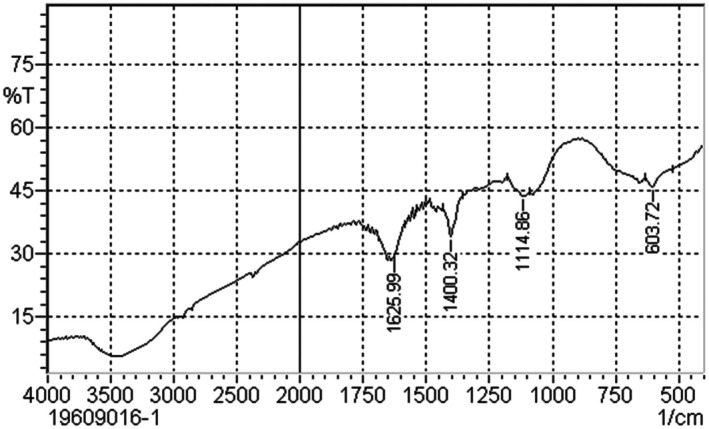
The FT‐IR wavelength spectrum of the leaf extract of *P. neriifolius* .

The characteristics of the compounds were disclosed based on their absorption spectrum, which includes infrared radiation. After the extract was processed using FT‐IR, the components' structural classifications were separated based on the peak ratio. The presence of the functional groups C=C, S‐S, C=O, CH2 bonds, and C‐C‐C bend was ensured as indicated in Figure 3 likely from steroidal glycosides, flavonoids, alkenes, aliphatic compounds, steroids, saponins, and glycosides.

### 3.3 In Vitro COX Inhibition Assay

The mean of the COX inhibition activity in the plant extracts was used to calculate the average COX‐1 and COX‐2 inhibition. A significant inhibitor of COX‐1 and COX‐2 was found in EEPN (mean activity COX‐1, 68.21% & COX‐2, 76.62%), with greater selectivity toward COX‐2 inhibition. Comparing the results with aspirin (1 mM), COX‐1 (11.33% ± 0.37%) and COX‐2 (14.21% ± 0.14%) were found.

### 3.4 Acute Oral Toxicity Test

Following an oral dosage of 2000 mg/kg, no behavioral alterations or mortality were noted, indicating that EEPN was not toxic to the experimental animals. The oral LD_50_ was therefore found to be more than 2000 mg/kg. Since the high dose for additional research was seen to be higher than 2000 mg/kg, 400 mg/kg (twice the highest tested dose) was used, and 200 mg/kg (one‐tenth of the highest evaluated dose) was designated as the low dose. As a result, the low dose for additional research was set at 200 mg/kg, which is one‐tenth of the highest tested dose, and the high dose was set at 400 mg/kg, which is twice the highest tested dose.

### 3.5 In Vivo Anti‐Inflammatory Activity

#### 3.5.1 Carrageenan‐Induced Mouse Paw Edema Method

**FIGURE 4:**
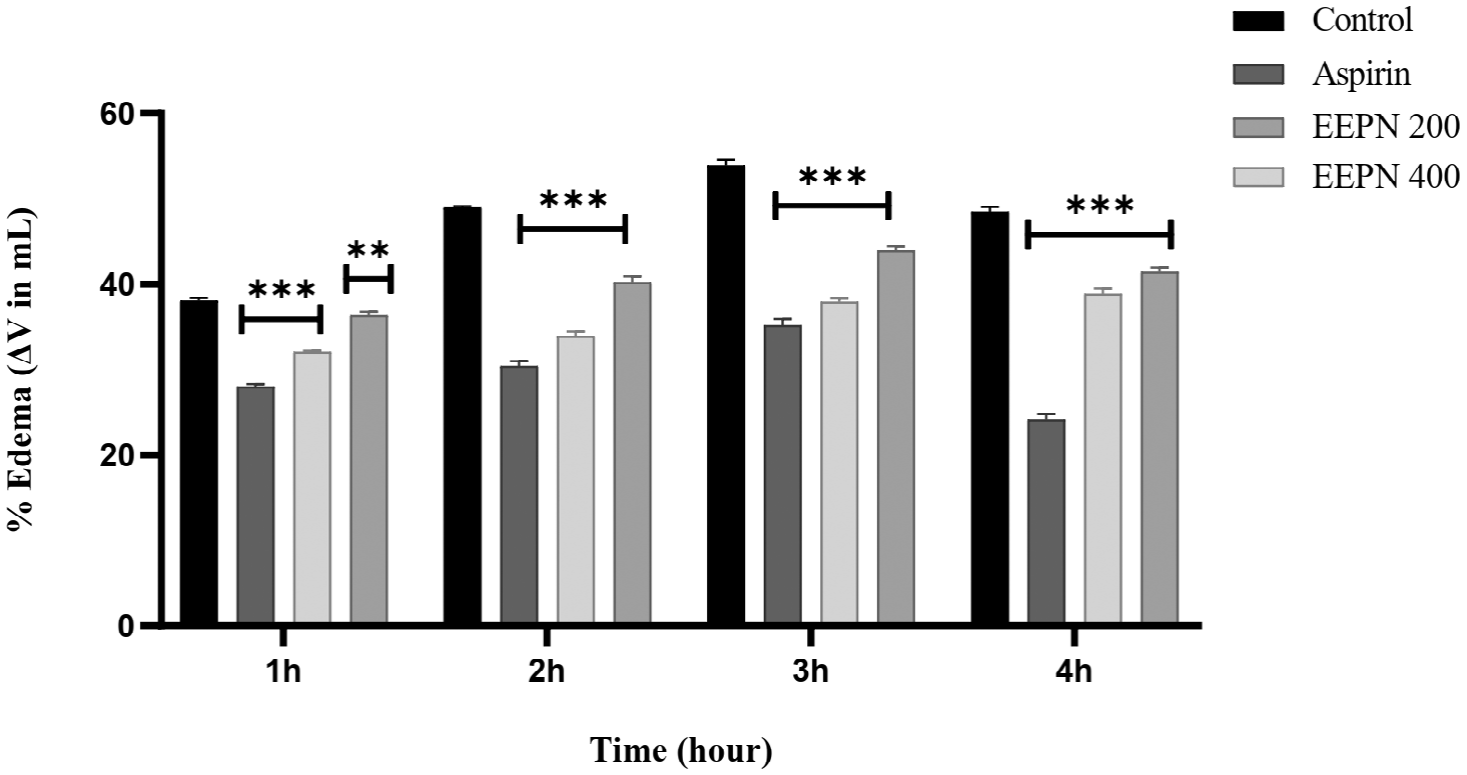
Anti‐inflammatory effects of EEPN are demonstrated as % Edema.

At 3 h, Carrageenan‐induced paw edema peaked. Edema was significantly decreased by ethanolic extract of *P. neriifolius* leaves (EEPN) (200, 400 mg/kg) (***p < 0.05), with 400 mg/kg exhibiting 38.88% ± 0.61% strong inhibition compared to aspirin's 24.12% ± 0.68% at 4 h, as shown in Figure 4.

### 3.6 Anxiolytic Profiling

#### 3.6.1 Elevated Plus Maze Method

**FIGURE 5:**
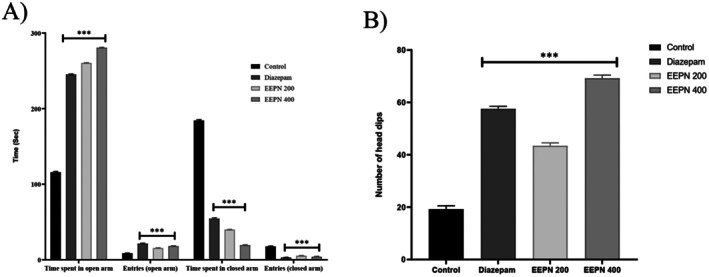
Anxiolytic effect of EEPN on mice through the elevated plus maze test (A) and hole board test (B). Data were expressed as Mean ± SEM (*n* = 5), and *p < 0.05, **p < 0.01, and ***p < 0.001 considered as significant. EEPN, ethanolic extract of leaves of *Podocarpus neriifolius* . Mean ± SEM (*n* = 5) is used to represent all values.

Ethanolic extract of *P. neriifolius* leaves (EEPN) (200 and 400 mg/kg) decreased closed arm time (39.8 and 19.3 s) compared to standard (54.4 s) and increased open arm time (260.3 and 280.6 s) compared to control (115.8 s) in the EPM test, shown in Figure 5A. There was notable anxiolytic activity at 400 mg/kg.

#### 3.6.2 Hole‐Board Test Method

Ethanolic extract of *P. neriifolius* leaves (EEPN) (400 mg/kg) exceeded the standard (57.5 ± 1.01) in the hole board test. It demonstrated highly significant anxiolytic activity (69.2 ± 1.22 head dipping, ***p < 0.001) in comparison to the control, shown in Figure 5B. There was little change at other doses, indicating that 400 mg/kg had a selective anxiolytic effect.

### 3.7 Sedative Profiling

#### 3.7.1 Hole‐Crossed Test

**FIGURE 6:**
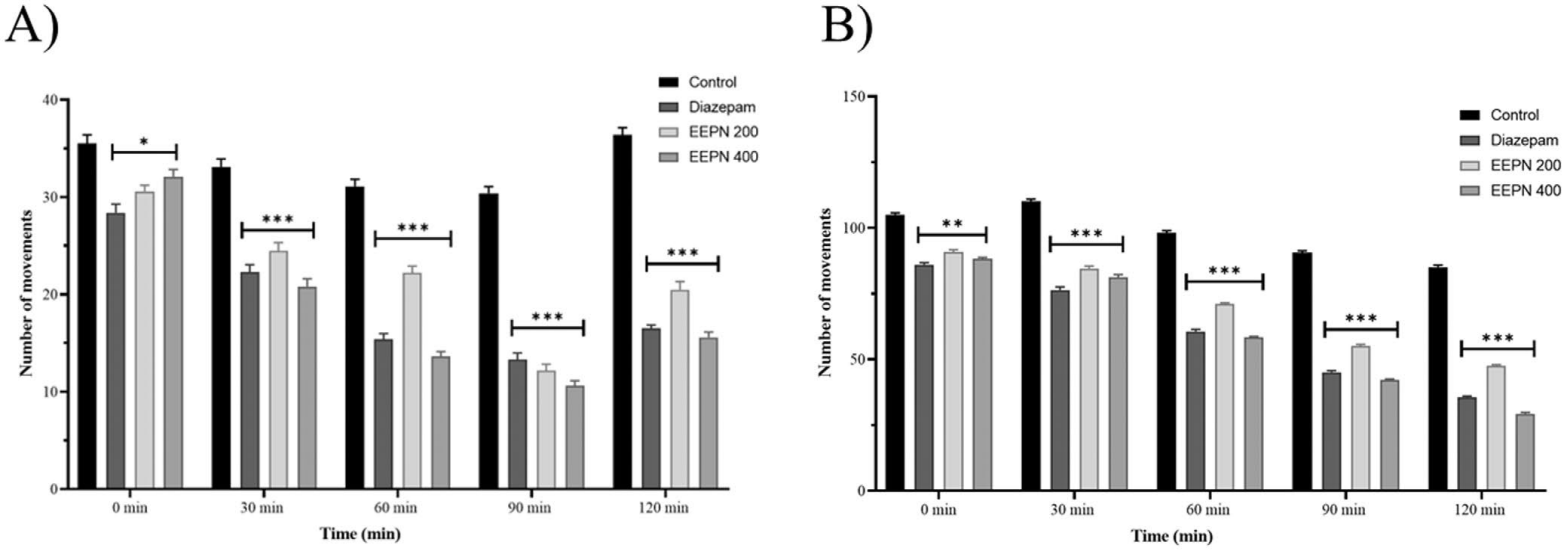
Sedative effect of EEPN on mice through the hole cross method (A) and open field test (B).

In comparison to the standard (13.3 ± 0.68 no. of movements), ethanolic extract of *P. neriifolius* leaves (EEPN) (200 and 400 mg/kg) significantly (***p < 0.001) decreased locomotor activity at 90 min (12.2 ± 0.64 and 10.6 ± 0.53 no. of movements), portrayed in Figure 6A, suggesting a sedative effect and less exploratory behavior.

#### 3.7.2 Open‐Field Test

Ethanolic extract of *P. neriifolius* leaves (EEPN) (400 mg/kg) markedly (***p < 0.001) decreased locomotor activity at 60 min (58.3 vs. control 98.2 no. of movements) and 120 min (29.2 vs. control 85.1 no. of movements), illustrated in Figure 6B with values below the standard, suggesting a possible sedative effect, according to open field data.

### 3.8 Antidepressant Activity

#### 3.8.1 Forced Swimming Test (FST) on Mice

**FIGURE 7:**
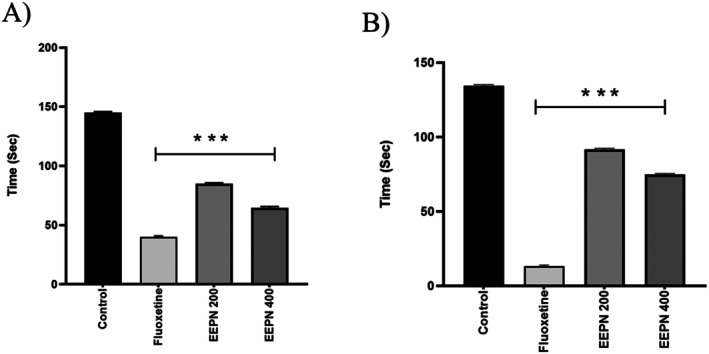
Antidepressant effect of EEPN on mice through the forced swimming test (A) and tail suspension test (B).

The immobility time (s) in the FST decreased as the dose increased, with this decrease being dose‐specific. The highest measures of immobility for EEPN 400 mg/kg (b.w.) were 64.66 ± 0.53 s (***p < 0.001). In addition, the standard drug (fluoxetine, 5 mg/kg b.w., i.p.) yielded an FST value of 39.87 ± 0.52 s (***p < 0.001), demonstrated in Figure 7A.

#### 3.8.2 Tail Suspension Test (TST) on Mice

The EEPN reduced immobility time in the TST experiment using a dose‐specific approach (***p < 0.001), a method used to estimate antidepressant activity. The TST immobility times were 92.21 ± 0.43 and 75.36 ± 0.42 (***p < 0.001) at the doses of 200 and 400 mg/kg, b.w., respectively, administered orally, as mentioned in Figure 7B.

### 3.9 Molecular Docking Outcomes

**FIGURE 8:**
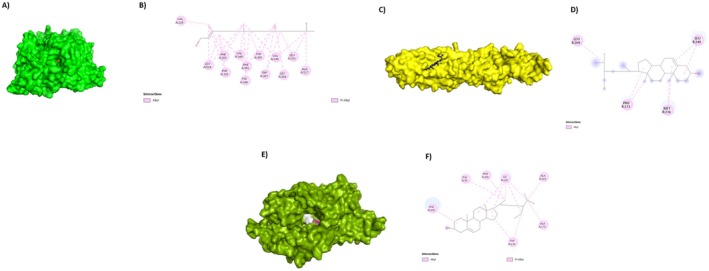
Interactions of human cyclooxygenase 2 with Phytol: (A) 3D and (B) 2D view. Interactions of Human γ‐aminobutyric acid type A (GABA_A_) with Gamma‐sitosterol: (C) 3D and (D) 2D view. Interactions of Human serotonin transporter with Gamma‐Sitosterol: (E) 3D and (F) 2D view.

Molecular docking predicts the binding orientation and energy of chemical compounds within three‐dimensional molecular targets to identify potential ligands. This essential tool facilitates the discovery of new therapeutic candidates through virtual screening while supporting structure‐based optimization and mechanistic studies [50, 51]. The molecular docking findings are presented in Table 3 for EEPN compounds with potential anti‐inflammatory, anxiolytic, and antidepressant properties, along with their corresponding binding affinities. Human Cyclooxygenase‐2 (PDB: 5IKR), Human Human γ‐aminobutyric acid type A (GABA_A_) receptor (PDB: 6X3T), and Human serotonin receptor (PDB: 5I6X) are among the targets. The top two EEPN compounds are compared with reference drugs (Standard drugs) to highlight their binding affinities and pharmacological potential. Docking scores are displayed in Table 3, and interaction details are shown in Tables 4, 5, 6 and Figures 8 (A‐F).

**TABLE 3:** Molecular docking results of GC–MS compounds against each protein.

Compound	Binding affinity		
	Anti‐inflammatory	Anxiolytic	Antidepressant
	5IKR	6X3T	5I6X
2R‐acetoxymethyl‐1,3,3‐trimethyl‐4 t‐(3‐methyl‐2‐buten‐1‐yl)‐1 t‐cyclohexanol	−4.7	−4.7	−8.2
Tetracontane‐1,40‐diol	−6.3	−3.2	−7.2
Phytol	**−6.6**	−3.8	−7
Decahydro‐8a‐ethyl‐1,1,4a,6‐tetramethyln	−3.4	−4.4	−7.5
Gamma‐sitosterol	−5.3	**−5.4**	**−10**
Standard (Aspirin/Diazepam/Fluoxetine)	−6.3	−5.1	−9

*Note:* The top‐docked compound with the highest binding affinity against each protein is presented in bold.

**TABLE 4:** Non‐bonds result from molecular docking for anti‐inflammatory properties against the top compounds and the standard drug for the Cox‐2 protein (PDB: 5IKR).

Protein ID	Compound name	Binding affinity (kcal/mol)	Bond types	Amino acid residue
5IKR	Phytol	−6.6	Alkyl	Val‐349 (2), Val‐344, Val‐228, Leu‐534 (3), Leu‐384, Leu‐352 (2), Ala‐527
			Pi‐Alkyl	Tyr‐385 (2), Tyr‐348 (2), Trp‐387 (2), Phe‐381 (2), Phe‐209 (2), Phe‐205 (3)
	Tetracontane‐1,40‐diol	−6.3	Conventional Hydrogen Bond	Arg‐513 (2), Glu‐524
			Carbon Hydrogen Bond	Tyr‐355, Glu‐524, Gln‐192
			Alkyl	Pro‐86 (2), Val‐89 (3), Val‐116 (3), Val‐349 (3), Leu‐352, Val‐523 (3), Ala‐527 (3), Leu‐531 (2), Leu‐359, Leu‐93, Leu‐352 (3), Met‐522
			Pi‐Alkyl	Tyr‐115, Tyr‐355 (3), Phe‐381, Tyr‐385, Trp‐387 (2), Phe‐518
	Aspirin	−6.3	Carbon Hydrogen Bond	Gly‐526, Ala‐527
			Amide‐Pi Stacked	Met‐522, Val‐523, Gly‐526, Ala‐527
			Pi‐Alkyl	Leu‐352, Val‐523, Ala‐527

#### 3.9.1 Molecular Docking for Anti‐Inflammatory Activity

COX‐2 was selected to examine the anti‐inflammatory potential of EEPN. The phytochemicals from EEPN showed significant binding affinities for COX‐2 (−6.6 to −3.4 kcal/mol). Phytol, the leading‐docked compound, showed −6.6 kcal/mol, where the standard drug showed affinity to −6.3 kcal/mol. Table 4 and Figure 8A,B summarize the results, covering the binding of both 3D and the highest‐scoring compound, as well as the standard drug, in both 3D and 2D formats.

#### 3.9.2 Molecular Docking for Anxiolytic Activity

To assess the anxiolytic properties of EEPN phytochemicals, GABA_A_ was chosen. These phytochemicals exhibited binding energies ranging from −5.4 to −3.2 kcal/mol for the Human GABA_A_ receptor. The highest binding affinity, −5.4 kcal/mol, was exhibited by Gamma‐sitosterol, which was greater than the reference drug (Diazepam), −5.1 kcal/mol. Table 5 summarizes the results, covering binding affinities, bond types, and amino acid residues. Figure 8C,D shows the highest‐scoring compounds and the standard drug in both 3D and 2D formats.

**TABLE 5:** Molecular docking for anxiolytic activity (PDB: 6X3T) against the top compounds and the standard drug produces non‐bonds.

Protein ID	Compound name	Binding affinity (kcal/mol)	Bond types	Amino acid residue
6X3T	Gamma‐sitosterol	−5.4	Alkyl	Pro‐233 (2), Met‐236 (2), Leu‐240 (2), Leu‐269
	2R‐acetoxymethyl‐1,3,3‐trimethyl‐4t‐(3‐methyl‐2‐buten‐1‐yl)‐1t‐cyclohexanol	−4.7	Conventional Hydrogen Bond	Gly‐224, Tyr‐225
			Carbon Hydrogen Bond	Asn‐189
			Alkyl	Ile‐228
			Pi‐Alkyl	Tyr‐225 (3)
	Diazepam	−5.1	Conventional Hydrogen Bond	Pro‐233
			Alkyl	Leu‐240
			Pi‐Alkyl	Met‐236, Leu‐240

#### 3.9.3 Molecular Docking for Antidepressant Activity

Table 6 summarizes the results, which include amino acid residues and bond categories. Figure 8E,F shows the top compounds and the standard drug in three‐dimensional and two‐dimensional representations.

**TABLE 6:** Molecular docking for antidepressant properties of lead compounds and standard drug against the target protein (PDB: 5I6X) yields non‐bonding interactions.

Protein ID	Compound name	Binding affinity (kcal/mol)	Bond types	Amino acid residue
5I6X	Gamma‐sitosterol	−10	Alkyl	Ala‐169, Ile‐172 (5), Ala‐173
			Pi‐Alkyl	Tyr‐95, Tyr‐176 (2), Phe‐335, Phe‐341
	2R‐acetoxymethyl‐1,3,3‐trimethyl‐4t‐(3‐methyl‐2‐buten‐1‐yl)‐1t‐cyclohexanol	−8.2	Conventional Hydrogen Bond	Phe‐335
			Carbon Hydrogen Bond	Gly‐338 (2)
			Pi‐Sigma	Tyr‐95
			Alkyl	Ile‐172 (2)
			Pi‐Alkyl	Tyr‐95, Tyr‐176 (2)
	Fluoxetine	−9.3	Conventional Hydrogen Bond	Asp‐98
			Carbon Hydrogen Bond; Halogen (Fluorine)	Ala‐173, Ser‐439
			Carbon Hydrogen Bond	Asp‐98, Ala‐96, Ser‐336, Tyr‐95
			Halogen (Fluorine)	Ile‐172, Ser‐439
			Pi‐Pi T‐shaped	Tyr‐176
			Alkyl	Ala‐173, Ile‐172
			Pi‐Alkyl	Tyr‐176, Ile‐172 (2), Val‐501

### 3.10 Molecular Dynamics Simulations

**FIGURE 9:**
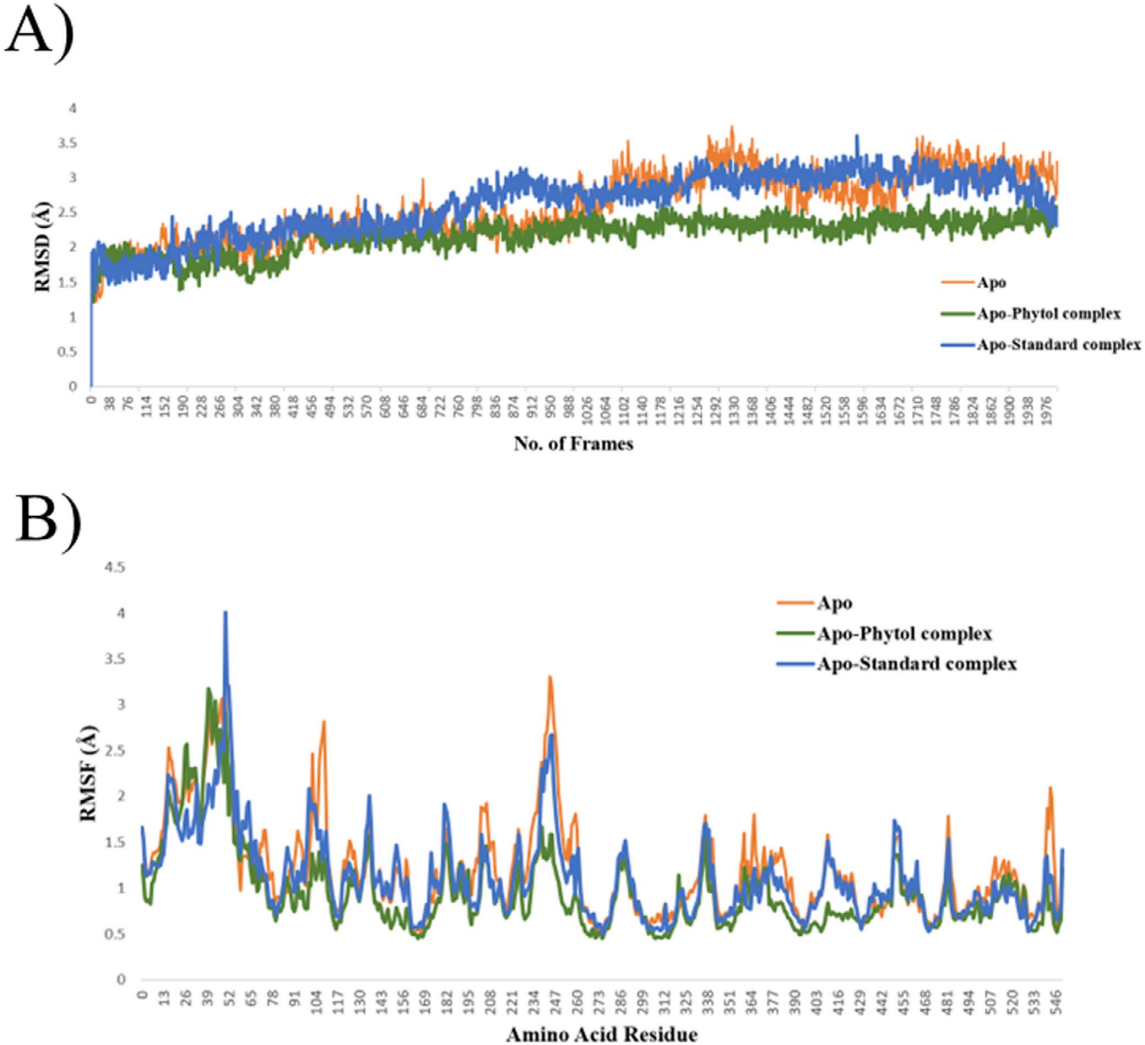
MD simulation results of Cox‐2 (Apo), Apo‐Phytol complex, and Apo‐Standard (Aspirin) complex. (A) RMSD, and (B) RMSF portray the flexibility and structural alterations of the three systems, representing color for Apo (orange), Apo‐Phytol complex (green), and Apo‐Standard complex (blue).

**FIGURE 10:**
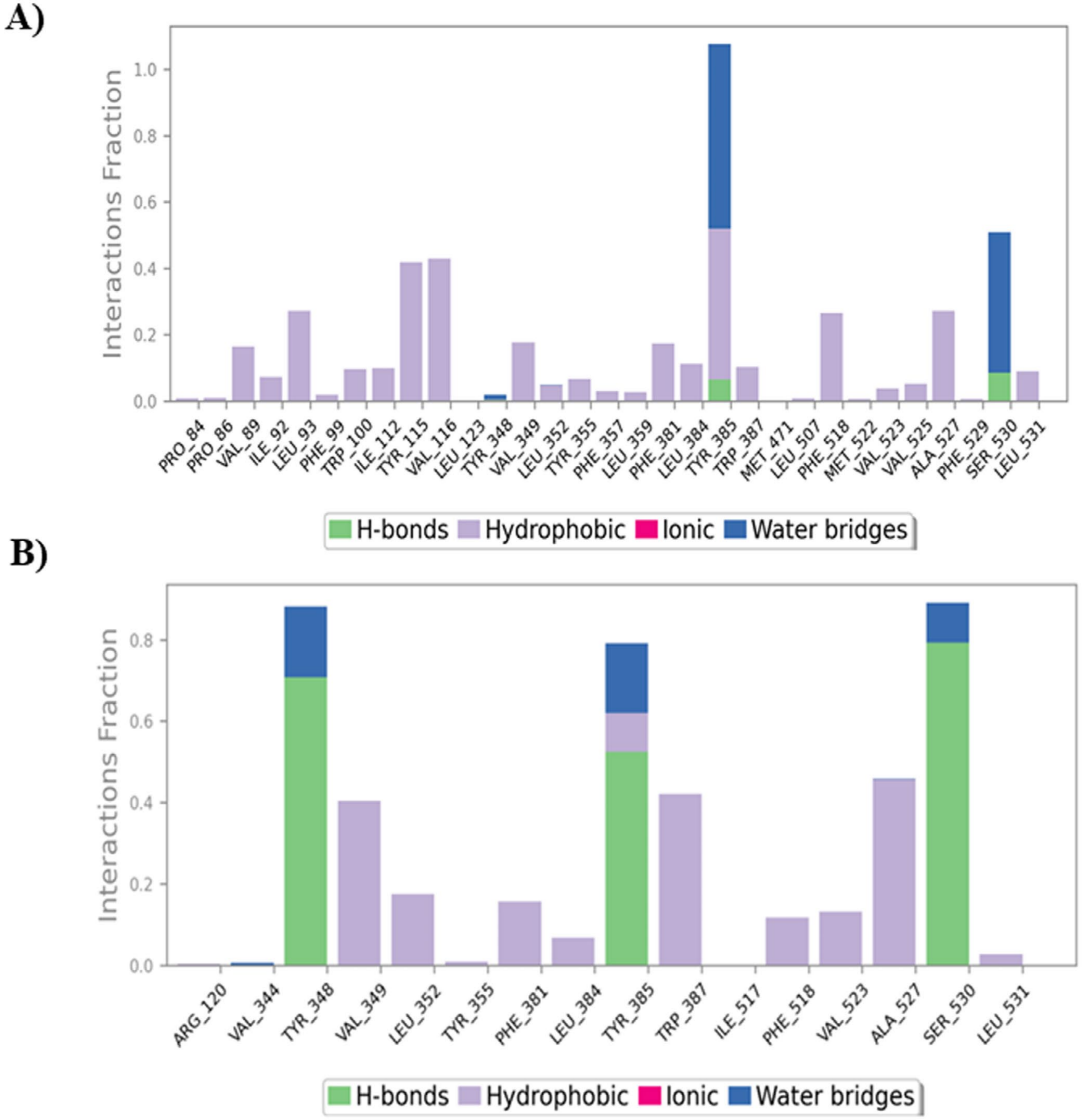
Interaction between Cox‐2 proteins and ligands, or the docked complex contact profile. Protein–ligand contacts of the following: (A) Apo‐Phytol complex, and (B) Apo‐Standard (Aspirin) complex. Green, purple, blue, and pink denote hydrogen bonding, hydrophobic interactions, water bridge interactions, and ionic bonding, respectively. The y‐axis shows the overall percentage of interactions between individual residues throughout the simulation.

**FIGURE 11:**
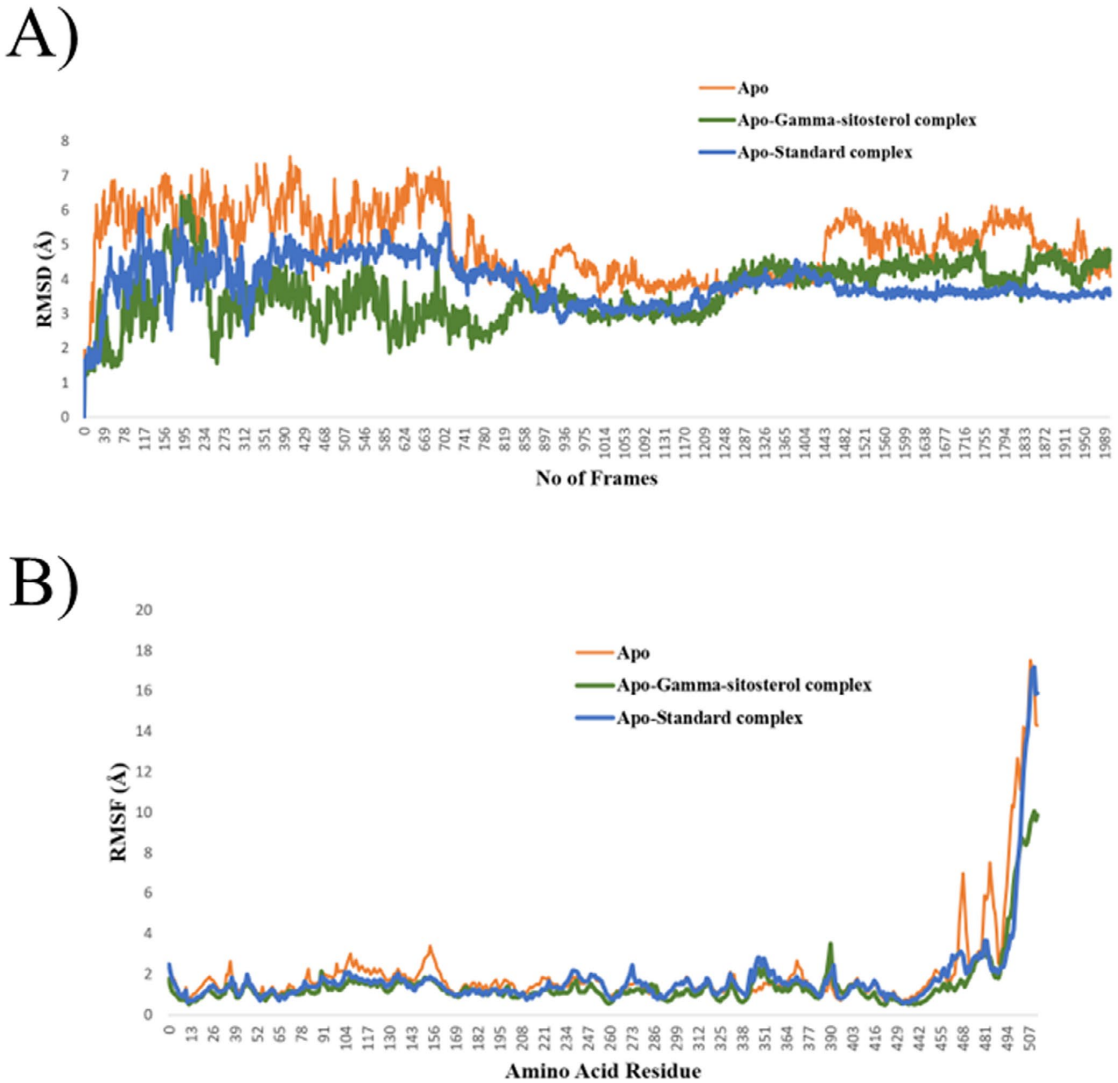
MD simulation results of Human GABA_A_ receptor (Apo), Apo‐Gamma‐sitosterol complex, and Apo‐Standard (Diazepam) complex. (A) RMSD, and (B) RMSF illustrate the structural alterations and flexibility of the three systems, representing color for Apo (orange), Apo‐Gamma‐sitosterol complex (green), and Apo‐Standard (Diazepam) complex (blue).

**FIGURE 12:**
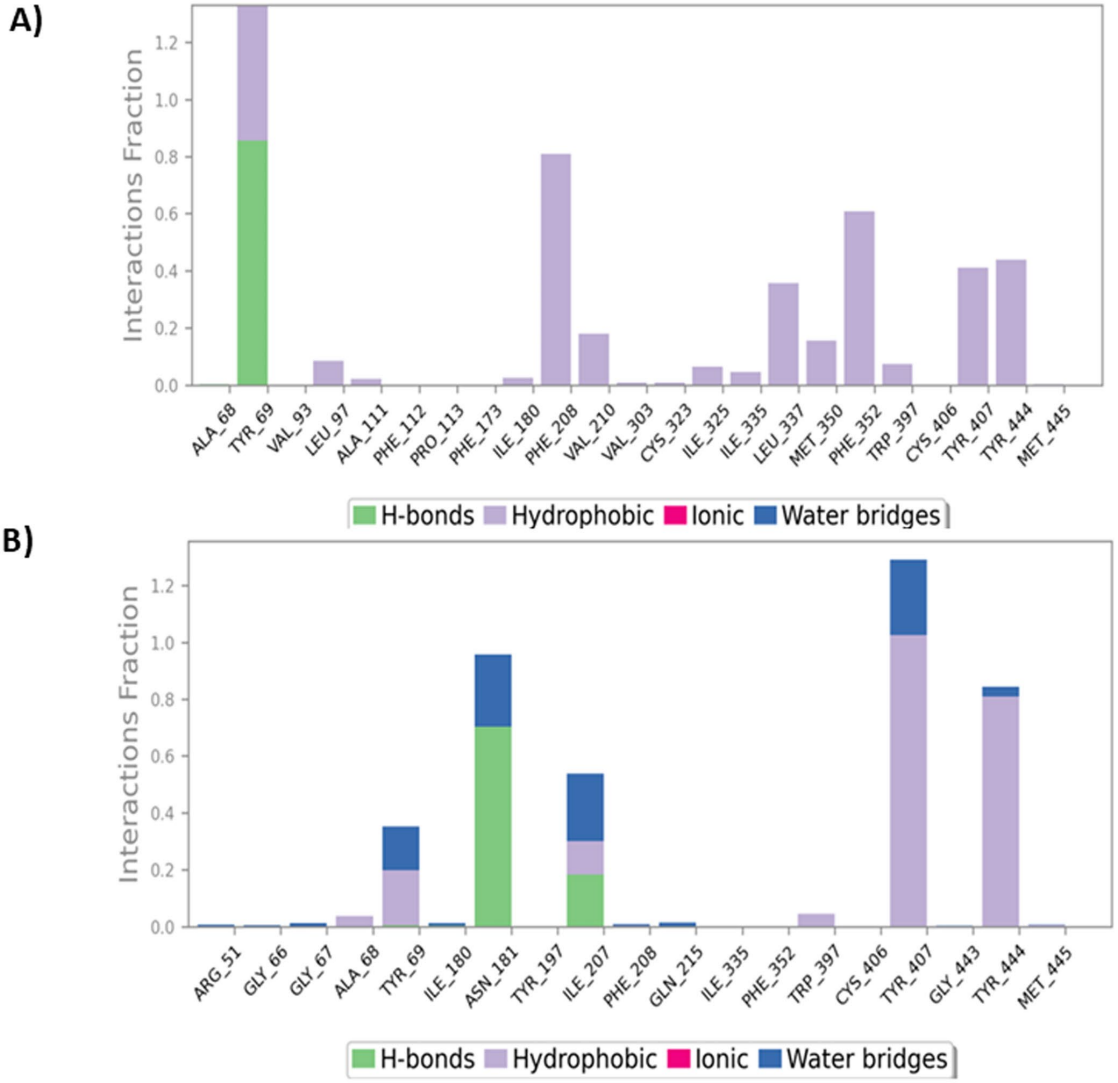
Interaction between Human GABA_A_ protein and ligands, or the top docked complex contact profile. Protein–ligand contacts of the following: (A) Apo‐Gamma‐sitosterol complex, and (B) Apo‐Standard (Diazepam) complex. Green, purple, blue, and pink demonstrate hydrogen bonding, hydrophobic interactions, water bridge interactions, and ionic bonding, respectively. The y‐axis shows the overall percentage of interactions between individual residues throughout the simulation.

In post‐docking analysis, MD simulation is essential because it enables the investigation of the robust and vibrant character of a large complex biological molecule over time. A 200 ns MD simulation for each complex was performed to learn more about the flexibility, binding mechanisms, and structural dynamics of the apo state of the Cyclooxygenase‐2 (COX‐2), the enzymes that make up the Human GABA_A_ receptor, and their complexes with Phytol, Gamma‐sitosterol, and their corresponding standard drugs. The computation and analysis of several parameters, including RMSD, RMSF, and protein‐ligand contacts, were performed based on a 200 ns dynamic trajectory. They were carried out since these evaluations are essential to obtaining desirable protein‐ligand stabilities. Figure 9 displays the graph resulting from the analysis of RMSD and RMSF, and Figure 10 shows the protein‐ligand contacts for the Cyclooxygenase‐2 enzyme. Again, Figure 11 displays the graph resulting from the analysis of RMSD and RMSF, and Figure 12 shows the protein‐ligand contacts for the Human GABA_A_ receptor.

#### 3.10.1 MD Simulation for Anti‐Inflammatory Activity

RMSD measures deviations in the protein backbone over time, reflecting structural stability during simulation [52]. The RMSD values for the Cα backbones in each of the three systems were calculated for a 200 ns simulation. All three systems eventually stabilize, as shown in Figure 9A. The Cox‐2 (apo) protein remained constant during the simulation; however, after 108 ns (1080 frames), a slight increase in RMSD was observed, which persisted until the end. With slight variations, the Apo‐Phytol complex exhibited comparatively stable behavior throughout the entire simulation. Conversely, it took 88 ns (880 frames) to reach total stability in the Apo‐Standard (Aspirin) complex, and it remained stable until 200 ns with intermittent minor variations. The Apo‐protein, Apo‐Phytol complex, and Apo‐Standard (Aspirin) complex had corresponding average RMSD values of 2.570, 2.168, and 2.617 Å. The Apo‐Phytol complex showed more stability than the apo protein and Apo‐Standard (Aspirin) complex, as evidenced by lower average deviations compared to the Apo and Apo‐Standard (Aspirin) complexes. As a result, this compound and the protein formed a stable complex.

RMSF analysis was used to identify flexible and rigid regions of the protein and to assess structural changes upon ligand binding [53]. The N‐terminus of the amino acid showed greater flexibility in all three systems (Figure 9B). Furthermore, the protein structures of all three systems showed observable flexibility at amino acid residues 36–50, 96–108, and 230–252. In contrast to the apo proteins (Cox‐2), both complexes exhibited noticeably less flexibility in the N‐terminal region of their protein structures. Additionally, the overall flexibility of this protein decreased upon ligand binding. Because this plant‐derived compound fits the protein's active site well, the Phytol interaction with the protein decreased flexibility and gave the protein's structure rigidity, indicating that stable complexes are forming.

To gain a better understanding of the creation and dissolution of new bonds, the ligand flexibility within the receptor's active site was also calculated. The total amount of protein‐ligand interactions was determined from the entire interaction matrix during the simulation phases to better clarify ligand selectivity and interaction patterns. As a result, Figure 10 displays that for every complex, the collective interaction among the ligand and protein includes the residual assistance in ligand binding. Despite the presence of other essential residues reported in the found Apo‐Standard (Aspirin) structure, the Apo‐Standard (Aspirin) complex showed, on average, very few hydrophobic and water bridge contacts. However, during the simulation trajectories in the Apo‐Standard (Aspirin) complex, Tyr‐348, Tyr‐385, and Ser‐530 showed a high number of hydrogen bonds. Figure 10A illustrates that the conserved residue Tyr‐385 accounted for the majority of the Phytol‐induced overall percentage of protein–ligand interactions by a considerable number of hydrogen, hydrophobic, and water bridge interactions. The RMSD and RMSF analyses may have predicted the strong interaction pattern observed between the Apo‐Phytol complex and the Cox‐2 binding site. It is known that the residues Tyr‐348, Val‐349, Leu‐352, Tyr‐355, Phe‐381, Leu‐384, Tyr‐385, Trp‐387, Phe‐518, Val‐523, Ala‐527, Ser‐530, and Leu‐531 are crucial for ligand recognition due to the strong hydrogen, hydrophobic, and polar interactions that Phytol mediates with them (Figure 10A,B). Curiously, conserved Tyr‐385 and Ser‐530 significantly enhanced the stability of Cox‐2 induced by Phytol, assuring the function of these residues in complex creation.

#### 3.10.2 MD Simulation for Anxiolytic Activity

For a 200 ns simulation, the RMSD values for the Cα backbones in each of the three systems were identified. As seen in Figure 11A, all three systems eventually stabilize at the end of the simulation. The Human GABA_A_ receptor (apo protein) remained unstable until the simulation reached 87 ns (870 frames). Still, after that, there was a significant decrease in RMSD that persisted until the end and remained stable. The Apo‐Gamma‐sitosterol complex remained unstable until the simulation reached 84 ns (840 frames), at which point the RMSD significantly declined and held stable till the very end. On the other hand, the Apo‐Standard (Diazepam) complex reached complete stability in 96 ns (960 frames) and remained stable till 200 ns, with occasional, slight fluctuations. The average RMSD values for the Apo‐protein, Apo‐Gamma‐sitosterol complex, and Apo‐Standard (Diazepam) complex were 5.03, 3.6, and 3.89 Å, respectively. Lower average deviations when compared to the Apo and Apo‐Standard (Diazepam) complex demonstrated that the Apo‐Gamma‐sitosterol complex was more stable than the Apo protein and Apo‐Standard (Diazepam) complex. Consequently, a stable complex was formed between this compound and the protein.

In all three systems, the C‐terminal amino acids exhibited greater flexibility (Figure 11B). Additionally, there was noticeable flexibility at amino acid residues 143–158 and 460–507 in the Human GABA_A_ protein structures of all three systems. Both complexes exhibited notably less flexibility in the C‐terminal region of the protein structures compared to the apo proteins. Since this compound interacts well with proteins, the generation of stable complexes was indicated by the Gamma‐sitosterol interaction with the protein, which decreased flexibility and imparted rigidity to the protein structure.

For each complex, the residual contribution to ligand binding and the total contact between the Human GABA_A_ protein and ligand are shown in Figure 12. In contrast to the Apo‐Gamma‐sitosterol complex, the Apo‐Standard (Diazepam) complex exhibited relatively few hydrophobic contacts, despite the presence of numerous other significant residues reported in the found Apo‐Standard (Diazepam) structure. However, during the simulation trajectories in the Apo‐Standard (Diazepam) complex, Asn‐181 and Ile‐207 revealed a large quantity of hydrogen bonds, whereas in the Apo‐Gamma‐sitosterol complex, Tyr‐69 showed a high number of hydrogen bonds. The strong interaction pattern observed between the Apo‐Gamma‐sitosterol complex in the Human GABA_A_ binding site may have been anticipated by the RMSD and RMSF analyses. It is known that the residues 108–118 and 210–216 are crucial for ligand recognition, as they surround the substrate/inhibitor cavity. The top compound, Gamma‐sitosterol, forms Ala‐111, Phe‐112, Pro‐113, and Val‐210 hydrophobic bonds with the protein. Both Apo‐Gamma‐sitosterol and Apo‐Standard (Diazepam) complexes formed interactions with Phe‐352, Trp‐397, Tyr‐407, and Tyr‐444, verifying the contribution of these residues in complex formation.

### 3.11 ADME/T Analysis

The pharmacokinetic and toxicity characteristics of the compounds found in EEPN by GC–MS analysis were evaluated using ADMET analysis (Table 7). Lipinski's Rule of Five was satisfied by every phytochemical except Tetracontane‐1,40‐diol found in EEPN, indicating good drug‐likeness and structural suitability for additional pharmacokinetic analyses. Their potential as oral active drug candidates is supported by this fundamental criterion. All other parameters of ADMET can fulfill the requirements for a drug compound.

**TABLE 7:** Phytochemical analysis using in silico ADMET of ethanolic extract of *Podocarpus neriifolius* leaves (EEPN).

Compound name	Absorption		Distribution		Metabolism	Excretion	Toxicity		Lipinski's rule of five	Bioavailability
	Water solubility (logmol/L)	Intestinal absorption (human) (%absorbed)	VDss (human) (logL/kg)	BBB permeability (logBB)	CYP3A4 substrate	Total clearance (logmL/min/kg)	AMES toxicity	Hepatotoxicity		
2R‐acetoxymethyl‐1,3,3‐trimethyl‐4t‐(3‐methyl‐2‐buten‐1‐yl)‐1t‐cyclohexanol	−3.774	93.319	0.148	0.275	No	1.209	No	No	Yes	0.55
Tetracontane‐1,40‐diol	−3.642	80.694	−0.678	−1.343	No	2.462	No	No	No	0.17
Phytol	−7.535	90.643	0.385	0.793	No	1.686	No	No	Yes	0.55
Decahydro‐8a‐ethyl‐1,1,4a,6‐tetramethyln	−6.91	94.886	0.648	0.786	No	0.99	No	No	Yes	0.55
Gamma‐sitosterol	−6.773	94.464	0.193	0.781	No	0.628	No	No	Yes	0.55

## 4 Discussion

Plant extracts may include substances with advantageous biological properties that form intricate combinations with multi‐target effects, concurrently blocking or regulating multiple important targets [54]. In this context, we investigate ethnomedicine in order to uncover the unexplored medicinal potential of *P. neriifolius* leaves, also referred to as the “Jati Brown pine.”

Carrageenan paw edema is an experiment frequently used to investigate anti‐inflammatory medications, because it involves multiple mediators, both steroidal and non‐steroidal [55]. Three stages are involved in the rat's local injection of Carrageenan, which causes an inflammatory response through the successive release of multiple mediators. Serotonin and histamine are discharged during the first 1.5 h [56], followed by a second period induced by bradykinin from 1.5 to 2.5 h, and a third period, whose messenger is thought to be prostaglandins, that takes place between 2.5 and 6 h following Carrageenan injection [57]. In terms of inflammatory processes, this third phase seems to be the most interesting. Thus, this third phase also marks the peak of the most pronounced vascular reflex, as measured by leukocyte transfer to the inflammatory area. It is commonly known that prostaglandins play a significant part in the inflammatory mechanism due to their ability to modulate inflammatory responses [58]. Rodents with carrageenan‐induced hind paw edema have been utilized to test the effects of NSAIDs, which primarily inhibit the COX‐producing prostaglandin synthesis. It has been shown that therapeutic dosages of the majority of clinically effective anti‐inflammatory drugs correlate reasonably with the reduction of hind paw edema brought on by Carrageenan after 3 h [58]. EEPN suppressed all three stages of carrageenan‐induced inflammation. This suggests that the active component or constituents may influence the production, discharge, and function of inflammatory messengers involved in edema induced by carrageenan. Thus, the present finding revealed that *P. neriifolius* has strong anti‐inflammatory activity. Our two lead compounds, Phytol (−6.6 kcal/mol) and Tetracontane‐1,40‐diol (−6.3 kcal/mol), exhibited a strong binding affinity for COX‐2 enzyme as compared to the standard drug Aspirin (−6.3 kcal/mol), determined by molecular docking analysis. By increasing catalase's stability, enzymatic activity, and resistance to degradation, these substances may improve catalase function. Thus, COX‐2 inhibition could be a possible mechanism underlying *P. neriifolius* leaves' anti‐inflammatory properties. Therefore, EEPN phytochemicals appear to possess promising and potent anti‐inflammatory properties, as evidenced by in vitro, in vivo, and molecular docking investigations.

The EPM and HBT, two popular behavioral models, were used to evaluate the anxiolytic effects of EEPN. Anxiolytic therapies usually encourage exploratory behavior, which increases the amount of time spent in the open arms and the number of entries [59]. EEPN significantly improved these parameters in our study, demonstrating its anxiolytic effects. Head dips are used in the HBT to measure anxiety levels, and anxiolytic medications increase this behavior [60]. The anxiolytic properties of EEPN (200 and 400 mg/kg) were further confirmed by the significant increase in head‐dip counts when compared to the control group. Benzodiazepines and barbiturates are two examples of frequently prescribed anxiolytics that work by altering GABAergic activity. The top‐docked compound, Gamma‐sitosterol, has a binding affinity of −5.4 kcal/mol, which exceeded the standard drug Diazepam −5.1 kcal/mol against the GABA_A_ receptor. According to our molecular docking investigation, the phytochemicals in EEPN have strong binding affinities for the GABA_A_ receptor, suggesting its potential use as a GABAergic anxiolytic. Natural sources of GABA_A_ modulators have demonstrated reliable anxiolytic effects without compromising overall locomotor function [61].

Two behavioral techniques, the open‐field method and the hole‐cross method, were used in this study to assess the CNS depressant activity of the ethanolic extract from *P. neriifolius* leaves. Medications like diazepam, the reference drug used in this experiment, decrease exploratory behavior that could have sedative effects and slow down the onset and duration of barbiturate‐induced sleep. The extract has strong sedative properties, according to both tests, as locomotor activity was significantly declined after administration of both 200 and 400 mg/kg b.w. doses (p.o.) as compared to the standard drug (Diazepam). GABA receptor is a potential target for a drug to show sedative activity [62]. According to the previously mentioned molecular docking analysis for the GABA_A_ receptor, phytochemicals from EEPN demonstrated a significant binding affinity, which denotes a possible sedative effect.

Two widely accepted procedures for forecasting the effectiveness of antidepressants are FST and TST. According to these studies, immobility, a characteristic behavior assessed in animals, is comparable to human depression. The drug's caliber to shorten mice's periods of immobility is equivalent to its antidepressant potential [63]. Animals that are exposed to unavoidably stressful situations show signs of despair or an incapacity to adapt, such as prolonged immobility. This behavior is very similar to human depression symptoms. The therapeutic potential of antidepressants is demonstrated by the reduction in immobility time it causes [59]. Compared with the control group, EEPN significantly reduced the duration of immobility in both test models at both tested doses. The serotonergic system is a significant target for antidepressant therapy since it is crucial for regulating mood, emotions, appetite, and sleep. Many clinically effective antidepressants target the serotonin transporter as their main target [64]. Phytochemicals in EEPN exhibit strong binding affinities for the serotonin transporter protein, as determined by molecular docking analysis. The highest binding affinity was demonstrated by Gamma‐sitosterol (−10 kcal/mol) in comparison to the standard drug Fluoxetine (−9.3 kcal/mol). This compound can penetrate the blood–brain barrier and have significant antidepressant effects.

To predict how atoms will behave in vibrant biological settings, molecular dynamics simulations are employed. According to MD simulation results, Phytol, a chosen hit, equilibrated following a 200 ns simulation period. Through the formation of multiple hydrogen bonds and hydrophobic interactions, residues Tyr‐385 and Ser‐530 preserved the stability of Phytol in the Cox‐2 protein–ligand contacts. This was noted in the RMSF analysis as well in Figure 9B. A 200 ns MD simulation was done for Gamma‐sitosterol, a chosen hit for the Human GABA_A_ protein. In the GABA_A_ protein–ligand interactions, residues Tyr‐69, Phe‐208, Phe‐352, and Tyr‐444 maintained the stability of Gamma‐sitosterol by creating multiple hydrogen bonds and hydrophobic interactions. The RMSF analysis also mentioned this in Figure 11B.

According to FT‐IR and GC–MS analysis, EEPN contains bioactive substances, including alkenes, glycosides, steroids, saponins, flavonoids, aliphatic compounds, and steroidal glycosides. Previous studies suggest that plants containing flavonoids, tannins, and saponins may have a beneficial effect on various central nervous system conditions [65].

The GC–MS technique is a valuable method for identifying bioactive substances in plant extracts [66]. GC–MS analysis of EEPN identified Phytol (RT 13.20 min; molecular weight 296; similarity index 86.0%) and Gamma‐sitosterol (RT 22.28 min; molecular weight 414; similarity index 78.7%) as prominent bioactive constituents. According to a study by Carvalho et al., Phytol, a diterpenoid derived from chlorophyll, had an anti‐inflammatory effect on mice, possibly through an interaction with the NFκB pathway [67]. The anxiolytic response can be facilitated by gamma‐sitosterol [68]. Costa et al. [69] found that Phytol, a diterpenoid derived from chlorophyll, had sedative and anxiolytic effects on mice, potentially through interaction with the GABAergic system. By decreasing immobility time in FST, another investigation revealed its antidepressant properties. The mouse brain also showed strong antioxidant effects [70]. A previously published study from Egypt reported the phytochemical profile of the methanolic extract of *P. neriifolius* , where diterpenoids and sterol‐related compounds were also detected, including dominant diterpene derivatives such as Kaur‐16‐ene [21]. In the present study, although ethanol was used as the extraction solvent instead of methanol, the identification of Phytol (a diterpenoid alcohol) and Gamma‐sitosterol (a phytosterol) indicates consistency in the occurrence of terpenoid and sterol‐based metabolites within the species. The variation in overall phytochemical composition between the two studies may be attributed to differences in solvent polarity, geographical origin, climatic conditions, and extraction methodology. The bioactive constituents identified in EEPN likely contribute to the pharmacological activities observed in this study. In addition, findings of molecular docking studies complement the observed pharmacological effects by suggesting potential mechanisms of action. Further, the molecular dynamics simulation study provides an in‐depth validation of our study. Moreover, phytochemicals of EEPN met Lipinski's rule of five, which denotes favorable drug‐like qualities, according to ADMET analysis. Additionally, their safety profile suggests that they might be suitable candidates for drugs.

Despite the traditional ethnomedicinal use of *P. neriifolius* , there has been a lack of systematic scientific investigation validating its pharmacological properties. The present study bridges this gap by providing the first integrated experimental and computational evaluation of *P. neriifolius* leaves. This study reports anti‐inflammatory, anxiolytic, sedative, and antidepressant potentials, as well as identifies promising bioactive compounds such as Phytol and Gamma‐sitosterol with significant interactions toward COX‐2, GABA_A_ receptor, and serotonin transporter proteins. Hence, this study establishes a scientific foundation for future bioassay‐guided fractionation, isolation, and structural characterization of active compounds. Further mechanistic studies, receptor‐binding assays, enzyme inhibition assays, and chronic toxicity evaluations will be necessary to validate these findings and explore their translational potential. Therefore, the present work contributes to natural product‐based drug discovery by identifying *P. neriifolius* as a promising source of multi‐target therapeutic candidates.

## 5 Conclusion

GC–MS analysis of the ethanolic extract of *P. neriifolius* leaves identified five compounds. EEPN demonstrated anti‐inflammatory, anxiolytic, sedative, and antidepressant effects both in vitro and in vivo. The compounds from EEPN exhibited strong binding affinities and stable behavior toward important drug target proteins, as determined by molecular docking and MD simulation studies. The compounds exhibited a favorable pharmacokinetic and safety profile, as indicated by ADMET studies. These results demonstrate the potential of EEPN as a phytomedicine for the treatment of illnesses like inflammation, anxiety disorders, major depressive disorder, and insomnia, where access to or side effects of existing treatments are a barrier. Further studies are needed to isolate active compounds, clarify mechanisms, and confirm safety to support clinical translation.

## Author Contributions

**Neamul Hoque:** conceptualization, data curation, formal analysis, investigation, methodology, visualization, writing – original draft. **Md. Tashrif Rahman Tipu:** investigation, writing – review and editing. **Md Abul Bashar:** investigation, writing – review and editing. **Raju Dash:** project administration, writing – review and editing. **Kutub Uddin Ahamed:** investigation, writing – review and editing. **Md. Liakot Ali:** investigation, writing – review and editing. **Md. Jahirul Islam Mamun:** investigation, writing – review and editing. **Bakul Akter:** investigation, writing – review and editing. **Mohammed Kamrul Hossain:** project administration, writing – review and editing. **Nawreen Monir Proma:** conceptualization, methodology, supervision, writing – review and editing.

## Funding

The authors have nothing to report.

## Conflicts of Interest

The authors declare no conflicts of interest.

## Supporting information


**Data S1:** Fourier transform‐infrared (FT‐IR) spectroscopy analysis.
Gas chromatography–mass spectroscopy (GC–MS) analysis.

## Data Availability

Data available on request from the corresponding author.
