# Supplementary material for: Investigating the Phytochemical Constituents, Anti‐Inflammatory, and Neuropharmacological Activities of Podocarpus neriifolius Leaves Through FT‐IR, GC–MS, Experimental Studies, Molecular Docking, and Molecular Dynamics Simulations
Source: Pharmacol Res Perspect. 2026 Jun 2;14(3):e70272. doi: 10.1002/prp2.70272 (PMC13238835; doi:10.1002/prp2.70272)
Supplement: Supplementary file 1 — Data S1: Fourier transform‐infrared (FT‐IR) spectroscopy analysis. Gas chromatography–mass spectroscopy (GC–MS) analysis. [file PRP2-14-e70272-s001.docx]

**Fourier transform-infrared (FT‑IR) spectroscopy analysis**

EEPN was placed at the KBr (FT-IR grade) pellet's exit point in order to perform the FT-IR analysis. Using a pellet press machine, these pellets were created from powdered KBr that had been pre-activated at 60°C for a full day. A Thermo Nicolet 6700 FT-IR spectrometer with an Xt-KBr beam splitter assembly and a DTGS-XT-KBr detector was used to record the extract's infrared spectra in the wavelength range of 4000–400 cm⁻¹. At a resolution of 4.0 cm⁻¹, each spectra was acquired over 32 scans.

**Gas chromatography-mass spectroscopy (GC-MS) analysis**

GC–MS analysis of the ethanolic extract of *P. neriifolius* leaves was performed using a SHIMADZU GCMS-QP2020 system equipped with an AOC-20s autosampler and AOC-20i autoinjector. Separation was carried out on a 30 m × 0.25 mm capillary column coated with 5% diphenyl–95% dimethyl polysiloxane. The injector temperature was maintained at 220 °C. The oven temperature program was initially set at 80 °C (held for 2 min), increased to 150 °C at a rate of 5 °C/min, and subsequently raised to 280 °C. Helium was used as the carrier gas at a constant flow rate of 1.72 mL/min. A 4 µL sample was injected with a split ratio of 1:100. Mass spectra were recorded in electron ionization (EI) mode at 70 eV, with a scan range of m/z 45–600 and a scan interval of 0.30 s during a total run time of 50 min. Compound identification was performed by comparing the obtained mass spectra with those available in the NIST 2008 and NIST 2014 mass spectral libraries integrated into the instrument software. Identification was confirmed based on spectral matching with a similarity index of ≥ 85%, along with careful examination of characteristic fragment ions and their relative peak intensities. The presence of major diagnostic fragment peaks and their consistency with reference spectra were considered for confirmation of compound identity. Only compounds meeting these criteria were reported.
